# Hydroponic and Aquaponic Floating Raft Systems Elicit Differential Growth and Quality Responses to Consecutive Cuts of Basil Crop

**DOI:** 10.3390/plants12061355

**Published:** 2023-03-17

**Authors:** Giuseppe Carlo Modarelli, Lucia Vanacore, Youssef Rouphael, Antonio Luca Langellotti, Paolo Masi, Stefania De Pascale, Chiara Cirillo

**Affiliations:** 1 Department of Agricultural Sciences, University of Naples Federico II, Via Università 100, 80055 Portici, Italy; 2 Centre for Innovation and Development in the Food Industry (CAISIAL), University of Naples Federico II, Via Università 100, 80055 Portici, Italy

**Keywords:** *Ocimum basilicum* L., circular economy, plant physiology, nutrients

## Abstract

Basil crops are appreciated for their distinct flavour and appeal to various cuisines globally. Basil production is mainly implemented in controlled environment agriculture (CEA) systems. Soil-less cultivation (e.g., hydroponic) is optimal for producing basil, while aquaponics is another technique suitable for leafy crops such as basil. Shortening the production chain through efficient cultivation techniques reduces basil production’s carbon footprint. While the organoleptic quality of basil demonstrably benefits from successive cuts, no studies have compared the impact of this practice under hydroponic and aquaponic CEA conditions. Hence, the present study evaluated the eco-physiological, nutritional, and productive performance of Genovese basil cv. Sanremo grown in hydroponic and aquaponic systems (combined with tilapia) and harvested consecutively. The two systems showed similar eco-physiological behaviour and photosynthetic capacity, which were on average 2.99 µmol of CO_2_ m^−2^ s^−1^, equal numbers of leaves, and fresh yields of on average 41.69 and 38.38 g, respectively. Aquaponics yielded greater dry biomass (+58%) and dry matter content (+37%), while the nutrient profiles varied between the systems. The number of cuts did not influence yield; however, it improved dry matter partitioning and elicited a differential nutrient uptake. Our results bear practical and scientific relevance by providing useful eco-physiological and productive feedback on basil CEA cultivation. Aquaponics is a promising technique that reduces chemical fertiliser input and increases the overall sustainability of basil production.

## 1. Introduction

Basil is a worldwide grown crop, particularly appreciated when fresh by customers due to its peculiar flavour, its richness in essential oils, and widespread adoption in the food, cosmetic, and medicinal industries [1,2]. In cuisine, basil is used as a spice to flavour pizza and pasta dishes and as a sauce in the form of pesto [3,4]. Generally, pesto production in the industrial sector requires a large number of plants to reduce the cost of production, and consecutive cuts are practiced to spare nursery costs and mechanise the harvest [5]. Currently, basil is mainly grown on soil or in hydroponics systems [6,7,8,9,10]. However, this production relies on intensive use of mineral fertilisers, with substantial environmental costs. The UN SDG and the farm-to-fork strategy aim to reduce water pollution and to protect marine and land life by reducing the waste of nutrients and the use of fertiliser [11,12]. To meet these goals, together with the increase in production costs (i.s. fertilizers) due to the post COVID-19 pandemic and the current war and with ongoing climate change, a shift to more sustainable and resilient food production systems has become pivotal [13]. Urban agriculture is a valuable means to shorten the production chain, create new job opportunities at the local level, and improve people’s overall quality of life [14,15,16,17,18]. Controlled environmental agricultural (CEA) systems are easily embedded into different urban scenarios, from abandoned greenhouses and open spaces to rooftop gardens and vertical farms [19,20,21,22,23,24,25]. Generally, CEA systems mainly require large inputs of mineral fertilisers [7]. However, an old but innovative technique is represented by aquaponics, which is the merger of aquaculture and hydroponic [25,26,27,28]. Aquaponics relies on fish feed as a nutrient input to produce both fish and plants, owing to the microbial action converting fish faeces, which are rich in ammonia, into available nitrogen for the plants with mutual benefit [29,30]. In fact, aquaponics allows aquaculture producers to reduce the waste of water and nutrients into the environment and the need for water and nutrients by plants [31].

Thanks to nitrate-rich water, aquaponics allows for the production of several leafy vegetables without the need for synthetic N-fertilizers. Among cultivated species, basil is the most commonly grown crop in aquaponics [32]. Basil production, especially for the food industry, requires high mechanisation to reduce labour costs and process vast amounts of product. Consecutive cuts have proved ideal for producing basil by diminishing the costs of plant materials and labour with benefits to yield and organoleptic qualities. However, most of the studies of the effects of consecutive cuts have been conducted in soil and hydroponic conditions [3,5,6,33], with few conducted in aquaponics under tropical environments [34].

To the best of our knowledge, no studies are available in the literature on the influence of consecutive cuts on basil growth and quality in hydroponic and aquaponics conditions in the Mediterranean climate.

For this reason, the study aimed to evaluate: (i) basil production, eco-physiological behaviour and nutrient profiles; and (ii) the influence of two consecutive cuts under aquaponics and hydroponic cultivation system conditions.

## 2. Results

### 2.1. Plant Growth

Basil plants under both cuts and both cultivation systems developed similar leaf numbers and total fresh biomass (Table 1). A significant interaction effect was found on the total leaf area. In fact, between the two cuts, the leaf area decreased by −57% in hydroponics, whereas it increased by 48% in aquaponics. The total dry weight and dry matter content at the first cut did not vary between the systems. In the second cut, the leaf area increased by 182% and 365% and by 166% and 208%, respectively, under hydroponic and aquaponic conditions, with higher values observed in plants grown in aquaponics compared to hydroponics. Morphological leaf traits were affected differently by the cut number and the type of system used. The specific leaf area (SLA) was greater in hydroponics under the first cut and decreased by −23% under the second, while it increased by 14% in aquaponics.

### 2.2. Gas Exchanges and Chl a Fluorescence Emission

Gas exchanges and *Chl a* fluorescence were affected differently by the cut number and the cultivation system used. Leaf net photosynthesis (P_n_), stomatal conductance (g_s_), transpiration (E), the maximal photochemical efficiency of the PSII (F_v_/F_m_), the actual yield of PSII (Φ_PSII_), and the electron transport rate (ETR) were higher under the first cut, and they decreased under the second cut. In contrast, the yield of non-regulated quenching (Φ_NO_) increased by +60% in the second cut, and the non-photochemical quenching did not vary. Concerning the cultivation system, plants grown under hydroponic conditions showed 14% higher transpiration and 25% ETR, while no differences were observed for the other measured parameters (Table 2). Compared with the first cut, the relative water content (RWC), the light use efficiency (LUE), and the intrinsic water use efficiency (WUE_i_) decreased in both systems on average by −122%, −567%, and −212%, respectively.

### 2.3. Mineral Content

The cut number and the cultivation system affected nutrient concentrations in leaf tissue differently (Table 3). Compared to the first cut, nitrate concentration of leaves harvested at the second cut increased by 1171% and 380% in hydroponic and aquaponics conditions, respectively (Table 3). Potassium did not vary between the two systems at the first cut, while it increased by 19% in hydroponic plants during the second cut (Table 3). Phosphorus was 38% higher in the first cut compared to the second one, and it was higher in hydroponics compared to aquaponics (Table 3). Magnesium concentrations were similar at the two cuts, and it was higher in aquaponics compared to hydroponics by 108% (Table 3). Leaf calcium concentration was higher in aquaponics than hydroponics, and compared to the first cut, it increased only in hydroponics (+104%) (Table 3). Chloride concentrations increased at the second cut on average by +133%, and it did not differ between the cultivation systems (Table 3). Sulphate and ammonia concentrations were higher in hydroponics than under aquaponics conditions. However, compared to the first cut, these concentrations increased only in hydroponics, with no differences in aquaponics between the two cuts. Lastly, sodium concentrations were not affected (Table 3).

### 2.4. Leaf Photosynthetic Pigments

Leaf photosynthetic pigment content was affected by both the number of cuts and the cultivation system. Compared to the first cut, the Chl a/b ratio increased in hydroponics by +35%, while it did not vary in aquaponics (Table 4). The total chlorophyll (a + b) and SPAD values decreased by −11% and −18% in hydroponics, respectively, while they did not vary in aquaponics. Carotenoid content was higher in aquaponics compared to hydroponics, and compared to the first cut, this content increased by 15% only in hydroponics plants (Table 4).

### 2.5. Cluster Heatmap Analysis

**Figure 1:**
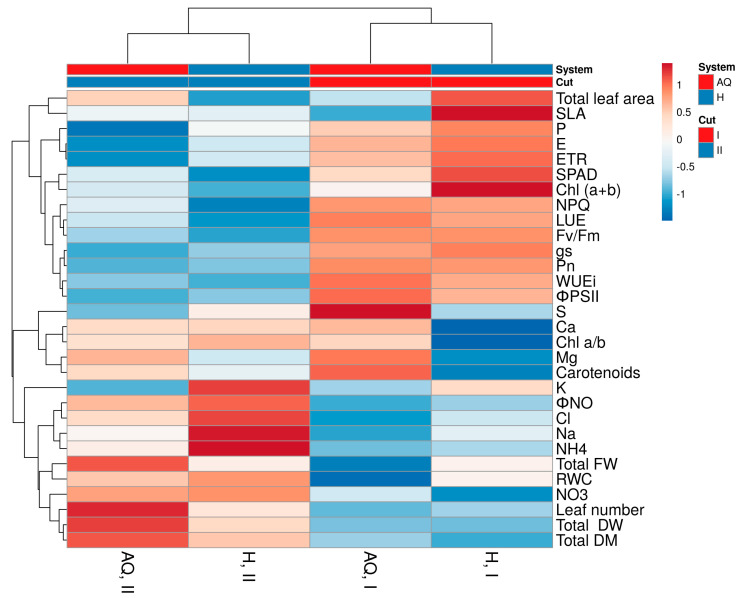
Heatmap cluster analysis in Genovese basil cv Sanremo plants under two consecutive cuts (35 (I) and at 79 (II) days after planting (DAP)) grown under hydroponics (H) or aquaponics (AQ) conditions. Original values are ln_(x+1)_ transformed; data are expressed with Euclidean distances and hierarchical clustering with complete linkage.

To obtain an in-depth overview of the morpho-anatomical, eco-physiological, and nutritional variations induced by the two cultivation systems and cut numbers, a cluster heatmap was created for all of the aforementioned parameters to highlight these differences better (Figure 1).

As a result, the main clustering factor was the cut number, rather than the cultivation system. Plants grown during the first cut were characterised mainly by a greater leaf area and higher leaf chlorophyll and carotenoid content, in line with higher SPAD values and higher maximal photochemical efficiency and the yield of the PSII, the LUE, and the WUEi. In addition, plants grown until the first cut absorbed more phosphorous. The accumulation of some nutrients, such as sodium, ammonium, and chloride, were reduced.

On the other hand, plants grown until the second cut showed higher leaf numbers and biomass accumulation, higher leaf NO_3_, K, Cl, NH_4_, and Ca content, and a higher non-regulated dissipation process in fluorescence. In addition, lower gas exchange activities, chlorophyll content, and fluorescence performance were observed.

To be more specific, plants grown under aquaponics conditions during the second cut showed the greatest increases in leaf number and fresh and dry biomass and lower P accumulation and gas exchange activity. During the first cut, aquaponics-grown plants showed the most elevated sulphate concentrations, the highest WUE_i_ and Φ_PSII_, and the lowest biomass production. In contrast, basil plants grown under hydroponic conditions during the first cut showed the greatest total leaf area, SLA, chlorophyll content, and SPAD values and the lowest concentrations of Ca, Mg, and Chl_(a/b)_ ratio. During the second cut, plants grown under hydroponic systems showed the highest NO_3_, K, Cl, Na, NH_4_, relative water content, and Φ_NO_ values and the lowest gas exchanges, chlorophyll fluorescence, and LUE (Figure 1).

## 3. Discussion

Identifying, developing, and optimising plant production more sustainably is a common goal to achieve the UN SDG and farm-to-fork strategy goals by 2030 [11,12,13].

Soilless cultivation of aromatic plants has been established worldwide. Hydroponic cultivation allows for successful growing of several vegetables and aromatic plant species, with high nutrient levels and water use efficiency. Aquaponics, on the other side, does not rely on chemical fertilisers, such as nitrates, owing to the nitrate-rich water produced by fish metabolism and converted by bacteria [35].

Basil on a commercial scale is consequently cut to spare resources, labour, and time; several studies in hydroponic conditions have shown improved fibre content, aroma profiles, and nutritional benefits [9].

To the best of our knowledge, this study is the first aiming to compare in a Mediterranean greenhouse environment the differences in growth, eco-physiology, and macro-nutrient concentrations of basil grown under hydroponics or aquaponics conditions under consecutive cuts since no studies seem to be available in the literature on these aspects.

Consecutive cuts in basil have been extensively evaluated in soil and substrate cultivation [3,5,6,9] and under hydroponic conditions [32] to improve the yield, photosynthetic performance, aromatic profile, polyphenols, and essential oil content [3]. In a tropical environment, other authors have provided information about the effects of consecutive cuts and periods of harvest on the yield [28] such that, after the fourth cut, the production decreased, suggesting harvesting a maximum of four times. In our study, we performed only two consecutive cuts, and the cut number predominantly impacted most of the analysed parameters, rather than the cultivation system used. However, the cultivation system influenced specifically the mineral content, such as of Mg. Saha and co-authors [36] compared basil productive performance in hydroponic and aquaponic systems with crayfish; however, they did not consider the cut effect, and a proper comparison was not possible due the different aquatic species used.

Generally, the cut in different vegetable species promotes growth thanks to the inhibitory effects of cuts on auxins, hence reducing the apical dominance; and by fostering cytokinin and gibberellin biosynthesis, it in turn allows new tissues and leaves to develop [3] and increases the node number and leaf number. In our experimental conditions, the cut number and the cultivation system did not affect the number of leaves. At the second cut, the leaf area decreased in hydroponic conditions while it increased in aquaponics. The fresh biomass did not vary between the two systems and the cuts. In contrast, most of the studies available in the literature reported an increase in fresh biomass [3,6,33]. In our research, in line with the previous study, the dry biomass and dry matter content increased between the first and the second cuts [3,6,33], in line with a higher RWC; in contrast, the SLA decreased in hydroponics, and it increased in aquaponics.

From a physiological perspective, there were no observed differences between the cultivation systems in P_n_ or fluorescence parameters, such as F_v_/F_m_ and Φ_PSII_. The cut number was the main factor. All eco-physiological parameters decreased in the second cut compared to the first, except for an increase in non-regulated dissipation processes such as Φ_NO_. This outcome is in line with the SPAD readings and photosynthetic pigment content. In fact, between the two cultivation systems, hydroponics showed higher chlorophylls, especially Chl a content, and lower carotenoid content. At the same time, aquaponic plants produced higher Chl b, as revealed by the Chl a/b ratio and carotenoids, compared to hydroponic plants. The chlorophyll content decreased between the first and second cuts only in hydroponics, in which we observed as a response an increase in carotenoid content to cope with possible photodamage and nutrient imbalances, such as in iron or manganese [37,38]. It is well known that light and other environmental variables directly affect gas exchanges and plants’ capacity to absorb light [39,40]. In our experiment, the leading cause of reductions in gas exchange in both systems has to be ascribed to the different environment and the particular light scenario since the first cut occurred in July and the second in September, with a large difference in light intensity and daylight integrals being reduced in September. The decrease in physiological activities during the second cut has to be ascribed to the combined effects of environmental changes, cut effects, and progressive tissue lignification, which occurs in plant tissue close to senescence with progressive degradation of RuBisCo activity and changes in the redox processes in photosystems [5,33]. In line with this hypothesis, apart from the reductions in photosynthetic and photochemical activities, as we previously stated, we recorded between the two cuts alterations in photosynthetic pigment composition and ratios in line with the decrease in the physiological parameters and the increases in Φ_NO_ and dry matter content, indicating the occurrence of a progressive plant’s tissue lignification.

On one side, hydroponic cultivation provides all the nutrients that plants need to develop and synthesise essential and beneficial compounds for human health [41]. However, it considerably impacts the environment since most nutrient sources, such as nitrates, comes from high-environmental impact processes. Phosphorus, in addition, is a finite and non-renewable element, and it is considered to be the next bottleneck that could cause our food system to collapse [42].

In fact, light can have a direct influence on nutrient uptake. Different studies have reported a species/variety-specific effect on nutrient uptake and concentrations in response to environmental and agronomic practices such as the cut number. In soil, basil experiments showed an increase in nitrate content [5] or a genotypic effect [3]. Studies in hydroponic conditions conducted between April and May 2019 reported decreases in nitrate content and phosphorous, potassium, calcium, and magnesium [33]. In our growing conditions, nitrate and ammonium concentrations increased in the second cut. At the same time, in agreement with the previous study, we reported a decrease in phosphorous, while calcium uptake increased only in hydroponics, and we did not observe differences in magnesium concentrations. As we previously stated, to account for the increase in nitrate content, the best hypothesis is the reduction in light intensity and DLI since light and temperature directly affect nitrate reductase and nitrate transformation in leaf tissue [43]. In fact, less nitrogen is used in the second cut for the primary and secondary metabolism since ammonium concentrations increased [44,45]. High nitrate content can be a serious threat to human health since it is implicated in human methemoglobinaemia disease [18]. EU regulation No 1258/2011 does not explicitly limit nitrate content for basil; however, considering the limit allowed for leafy vegetables, in our growing condition, we were less than that limit in both systems and cut periods [46]. In agreement, our results align with the nitrate content values observed by Ciriello, Formisano, Corrado and Nicoletto [3,5,6,9,33].

Furthermore, minerals are essential building blocks for crucial molecules and involve different pathways. Nutrient imbalances can cause impairment of metabolic functions and decreases in photosynthetic and water efficiency. In our study, we observed a substantial reduction in F_v_/F_m,_ close to photoinhibition in plants grown during the second cut, especially under hydroponic conditions, in line with a decrease in Φ_PSII_ and an increase in Φ_NO_, indicating high stress on the photosystems [47]. This outcome is ascribed mainly to a phosphorous deficiency since the P leaves’ concentrations decreased in the second cut. P is an essential component of molecules such as ATP and NADPH and is involved in several metabolic pathways, such as the electron transport chain between the PSII and PSI [42,48,49].

Further experiments should focus on the need to better understand the influence of the cultivation system on basil aroma and essential oil profile changes in relation to the cut number and the light intensity.

## 4. Materials and Methods

### 4.1. Experimental Site

The experiment was performed in a recirculating aquaponics system (RAS) prototype and floating hydroponic raft system inside a cold greenhouse (40°48′57.9″ N 14°21′01.6″ E, 29 m a.s.l.), shaded with a black net at 75% in the Department of Agricultural Sciences of the University of Naples Federico II (Portici, Italy) from 24 June to 10 September 2021. The RAS unit consisted of 4 rearing tanks, each of 2800 L, and tilapia fish (*Oreochromis niloticus*) of 4 weight classes were farmed at a mean stoking density of 8.7 ± 5.4 kg m^−3^. The system was equipped with an 800-L Superbead system for mechanical and biological filtration (Air-aqua, Wethouder Ohmannstraat, Staphorst, the Netherlands), a 400-L trickling filter (Scubla srl, Udine, Italy), and a 40-W UV sterilisation unit (Air-aqua, Wethouder Ohmannstraat, Staphorst, the Netherlands). Ambient air insufflation was set in the tanks at 0.05 v v^−1^ min ^−1^. The floating raft units, each 2 m^2^ in area in the case of the aquaponics treatment (AQ), were connected by 1 loop to the RAS unit, while in the hydroponics treatment (H), it was disconnected from the RAS unit and monitored separately.

### 4.2. Plant Material and Experimental Conditions

Two-week-old seedlings of Genovese basil, *Ocimum basilicum* L. cv. Sanremo, grown on polystyrene sowing trays, were used as plant material. The roots were gently washed with tap water to remove the peat cube and planted into a floating raft system of the RAS and hydroponic units, respectively, at a plant density of 20 plants per m^−2^. The water temperature was set to 23 °C, and its pH and electrical conductivity (EC) were monitored daily over the entire period and were, on average, 6.9 and 1100 µS cm^−1^, respectively

### 4.3. Aquaponic and Hydroponic Nutrient Solution Management

The RAS unit was fed with preformulated fish feed (42% protein content) (Tilapia Grower 13-EF, Alltech Coopens, Helmond, the Netherlands). The daily fish feed target was adjusted based on fish weight and stocking; during the experiments, the average daily feed intake was 1.4 ± 0.5% body weight. The hydroponic nutrient solution consisted of a half-strength Hoagland nutrient solution with a pH of 6.9 and an EC of 1100 µS cm^−1^. The concentrations of macro and meso nutrients of both systems are reported in Table S1. In each system the water temperature, pH, and EC were monitored daily (Thermo Scientific Expert pH and Cond Testers, Segrate, Italy). The hydroponics nutrient solution was reintegrated every second week, and the pH was adjusted with nitric acid or potassium hydroxide (KOH).

### 4.4. Plant Yield and Growth Measurements

Plant growth was determined during 2 consecutive cuts, at 35 and 79 days, respectively, after planting (DAP) on 18 plants per cut number × cultivation system. Cuts were performed with scissors leaving 2 buds below. The leaf number was recorded. The total leaf area was obtained by analysing digital images with ImageJ software, version 1.50i (Wayne Rasband National Institute of Health, Bethesda, MD, USA). Fresh weights of canopy and roots were recorded with an electronic balance, and dry weights were obtained after drying samples at 70 °C for 48 h.

### 4.5. Specific Leaf Traits

Specific leaf area (SLA) and relative water content (RWC) were determined on 3 fully expanded leaves per plant on 18 plants per cut number × cultivation system. SLA was calculated as the ratio between the single leaf area and its dry weight recorded after drying samples at 70 °C for 48 h. RWC was determined considering fresh leaf weight, turgid weight (after overnight with distilled water), and leaf dry weight.

### 4.6. Gas Exchanges and Chl a Fluorescence Emission

Gas exchange measurements were performed at 35 and 79 days after planting (DAP) on 1 fully expanded leaf from 6 plants × 3 replicates × cut number × cultivation system using a photosynthesis yield analyser (LCi T, ADC Bioscientific Ltd., Hoddesdon, UK); measurements were performed at noon in ambient CO_2_ (434 ppm) at a mean temperature of 31.1 °C and relative humidity of 45%, and a photosynthetic photon flux density (PPFD) of 1251.8 µmol m^−2^ s^−1^. Light use efficiency (LUE) was calculated as the ratio of the leaf net photosynthesis (P_n_) to the incident number of photons, and intrinsic water use efficiency (WUE_i_) was calculated as Pn/g_s_.

On the same leaves used for gas exchange measurements, chlorophyll *a* fluorescence emission was determined using a portable fluorimeter kit (Plant stress Kit, Opti-Sciences, Hudson, NY, USA). Measurements in the light were performed with a ΦPSII meter by applying a saturating pulse of 4286 µmol m^−2^ s^−1^ for 1.1 s, to obtain the maximum light-adapted fluorescence (F_m_′) and steady-state fluorescence (F_s_). For measurements in the dark, leaves were dark-adapted for 30 min with a dark leaf clip (Opti-Sciences Inc., Hudson, NY, USA); then, using an F_v_/F_m_ meter (Opti-Sciences Inc., Hudson, NY, USA), a 1.0-s saturating pulse light (3429 µmol m^−2^ s^−1^) was used to obtain the F_m_ and F_o_ values. The PSII maximum photochemical efficiency (F_v_/F_m_) was calculated as F_v_/F_m_ = (F_m_ − F_0_)/F_m_. The quantum yield of PSII electron transport (Φ_PSII_) was calculated as Φ_PSII_ = (F_m_′ − F_s_)/F_m_′ following Genty et al. [50].

### 4.7. Leaf Chlorophyll and Carotenoids Content and SPAD Index Determination

At each cut (35 and 79 days after planting (DAP), SPAD index and leaf photosynthetic pigment content were determined on 1 fully expanded leaf per 6 plants × 3 replicates × cut number × cultivation system. SPAD measurements were performed with a portable chlorophyll meter SPAD-502 (Konica Minolta, Tokyo, Japan). Leaf samples were immediately frozen at −20 °C until analysis. An aliquot of 0.5 g of leaf tissue was ground with 5 mL of acetone (80%) into a 15-mL tube flask. The solution was incubated in the dark at room temperature for 15 min, followed by 5 min of centrifugation at 3000× *g.* Pigment content was determined by light absorbance at 662, 645, and 470 nm for chlorophyll a, b, and total carotenoids, respectively, using a Hach DR 2000 spectrophotometer (Hach Company, Loveland, CO, USA). Total chlorophyll was calculated as the sum of chlorophyll a and b according to Lichtenthaler et al. [51].

### 4.8. Leaf and Stems Mineral Content Analysis

According to the protocol from Pannico et al. [52], a 250-mg aliquot of a ground-milled (model MF10.1, IKA-Werke GmbH & Co. KG, Staufen, Germany) dry leaf sample was used for the determination of leaf mineral (NO_3_, P, K, Ca and Mg) composition. Mineral analysis was then performed after 0.45-µm filtering using an ion chromatographer (model ICS-3000, Dionex, Sunnyvale, CA, USA) and quantified using an electrical conductivity detector equipped with IonPac CS12A and IonPac AS11-HC analytical columns for the analysis of cationic and anionic contents, respectively (Dionex, Sunnyvale, CA, USA). Except for nitrate, which was expressed on a fresh weight (FW) basis considering the leaves’ dry matter content, all the minerals leaf concentrations are expressed as g kg^−1^ on a dry weight (DW) basis.

### 4.9. Statistical Analysis

The experiment and all the analysis and statistics were conducted on an average of 6 plants × 3 replicates × cut number × cultivation system. Analysis of variance was performed using the SPSS software package, version 27 (IBM, Chicago, IL, USA). Means were compared by Tukey’s HSD post hoc test (*p* < 0.05). A cluster heatmap was generated using ClustVis online software [18] using Euclidean distance as the similarity measure and hierarchical clustering with complete linkage considering the cut number and the cultivation system. Values were log_(x+1)_ transformed.

## 5. Conclusions

To conclude, to develop sustainable food systems, food producers need to rely less and less on mineral fertilisers to improve the resilience of food systems. In our growing conditions, aquaponic and hydroponic cultivation seems to provide equal production. The cut and the period when it was performed influenced basil plants grown under both systems similarly and increased dry biomass and matter content. In addition, the systems promoted nitrate accumulation, mainly due to environmental changes between one cut and the others. Overall, both systems are a valuable means to produce basil in controlled environment agricultural systems, and aquaponics is highly suggested to improve the sustainability of the food sector. However, proper life cycle assessments must be performed to better quantify the impacts on different sustainability indicators.

In conclusion, our study brings new knowledge on the productive, eco-physiological behaviours and nutrient profile of basil grown in hydroponic and aquaponics systems. In addition, we report for the first time the cut number’s influence on basil plants grown under aquaponics conditions. Our results have practical and scientific relevance.

## Figures and Tables

**Table 1:** Plant growth in terms of leaf number, total leaf area, total fresh weight (FW), total dry weight (DW), total dry matter (DM) and specific leaf area (SLA) in Genovese basil cv Sanremo plants under two consecutive cuts (35 (I) and at 79 (II) days after planting (DAP)), grown under hydroponic (H) or aquaponics (AQ) conditions.

Treatments		Leaf Number	Total FW	Total Leaf Area	Total DW	Total DM	SLA
System (S)	Cut (C)	(n plant^−1^)	(g FW plant^−1^)	(cm² plant^−1^)	(g DW plant^−1^)	(%)	(cm² g)
H	I	36.12 ± 2.89	38.05 ± 1.67	2795.9 ± 133.19 a	1.16 ± 0.07 c	2.98 ± 0.06 c	411.3 ± 1.18 a
	II	46.67 ± 3.49	38.75 ± 2.83	1206.26 ± 147.76 c	3.26 ± 0.27 b	7.91 ± 0.13 b	315.7 ± 12.4 b
	Mean	41.39	38.4	2001.08	2.21	5.44	363.5
AQ	I	33.33 ± 0.71	32.36 ± 1.23	1476 ± 134.07 c	1.18 ± 0.03 c	3.64 ± 0.04 c	280.4 ± 0.007 c
	II	62.61 ± 4.17	44.38 ± 4.44	2186.86 ± 7.36 b	5.5 ± 0.37 a	11.22 ± 0.11 a	320.2 ± 0.72 b
	Mean	47.97	38.37	1831.43	3.34	7.43	300.2
Significance							
S		0.723 ^ns^	0.995 ^ns^	0.195 ^ns^	0.023 *	0.00 **	0.000 **
C		0.167 ^ns^	0.231 ^ns^	0.006 **	0.00 **	0.00 **	0.002 **
S × C		0.969 ^ns^	0.282 ^ns^	0.00 **	0.025 *	0.00 **	0.00 **

Data represent the mean ± s.e. (n = 3). Different letters indicate significance based on Tukey’s HSD post hoc test. ns, *, **, and *** indicate non-significant and significant effects at *p* < 0.05, 0.01, and 0.001, respectively.

**Table 2:** Gas exchanges and chlorophyll fluorescence emission in terms of leaf net photosynthesis (P_n_), stomatal conductance (g_s_), leaf net transpiration (E), maximal photochemical efficiency of PSII (F_v_/F_m_), yield of PSII (Φ_PSII_), linear electron transport rate (ETR), yield of non-regulated quenching (Φ_NO_), non-photochemical quenching (NPQ), intrinsic water use efficiency (WUEi), light use efficiency (LUE), and relative water content (RWC) in Genovese basil cv. Sanremo plants under two consecutive cuts (35 (I) and at 79 (II) days after planting (DAP)) grown under hydroponic (H) or aquaponics (AQ) conditions.

Treatment	P_n_	g_s_	E	F_v_/F_m_	Φ_PSII_	ETR	Φ_NO_	NPQ	WUE_i_	LUE	RWC
	(µmol CO_2_ m^−2^ s^−1^)	(mol m^−2^ s^−1^)	(mol H_2_O m^−2^ s^−1^)			(µmol m^−2^ s^−1^)			(µmol CO_2_ m^−2^ s^−1^/mol H_2_O m^−2^ s^−1^)	(µmol CO_2_ m^−2^ s^−1^/µmol photons m^−2^ s^−1^)	(%)
Cultivation system (S)											
H	2.97 ± 0.87	0.13 ± 0.01	3.36 ± 0.32 a	0.71 ± 0.05	0.42 ± 0.03	32.68 ± 4.79 a	0.21 ± 0.02	1.19 ± 0.13	20.96 ± 4.32	0.009 ± 0.004 a	92.46 ± 1.55
AQ	3.01 ± 0.98	0.12 ± 0.01	2.93 ± 0.35 b	0.73 ± 0.04	0.43 ± 0.04	25.99 ± 4.95 b	0.25 ± 0.04	1.36 ± 0.21	22.08 ± 5.23	0.013 ± 0.003 a	88.77 ± 2.22
Cut (C)											
I	4.93 ± 0.45 a	0.15 ± 0.01 a	3.85 ± 0.15 a	0.82 ± 0 a	0.51 ± 0.02 a	39.53 ± 2.83 a	0.2 ± 0.01 b	1.47 ± 0.09 a	31.80 ± 1.84 a	0.02 ± 0.001 a	87.75 ± 2.23 b
II	1.05 ± 0.06 b	0.09 ± 0.00 b	2.44 ± 0.14 b	0.62 ± 0.02 b	0.35 ± 0.01 b	19.15 ± 1.56 b	0.33 ± 0.02 a	1.07 ± 0.20 b	11.24 ± 3.78 b	0.003 ± 0.002 b	93.48 ± 0.66 a
Significance											
(S)	0.9403 ^ns^	0.423 ^ns^	0.029 *	0.263 ^ns^	0.694 ^ns^	0.039 *	0.378 ^ns^	0.483 ^ns^	0.599 ^ns^	0.013 *	0.115 ^ns^
(C)	0.00 **	0.00 **	0.00 **	0.00 **	0.00 **	0.00 **	0.002 **	0.123 ^ns^	0.00 **	0.00 **	0.025 *
CS × C	0.70 ^ns^	0.851 ^ns^	0.65 ^ns^	0.301 ^ns^	0.291 ^ns^	0.871 ^ns^	0.863 ^ns^	0.581 ^ns^	0.00 **	0.12 ^ns^	0.291 ^ns^

Data represent the mean ± s.e. (n = 3). Different letters indicate significance based on Tukey’s HSD post hoc test. ns, *, **, and *** indicated non-significant and significant effects at *p* < 0.05, 0.01, and 0.001, respectively.

**Table 3:** Leaf mineral concentrations, expressed for nitrate as g kg^−1^ f.w. and for all others as g kg^−1^ d.w., in Genovese basil cv. Sanremo plants in two consecutive cuts (35 (I) and at 79 (II) days after planting (DAP)) grown under hydroponics (H) or aquaponics (AQ) conditions.

Treatment		NO_3_	P	K	S	Ca	Mg	NH_4_	Na	Cl
System (S)	Cut (C)	(g kg^−1^ f.w.)	(g kg^−1^ d.w.)	(g kg^−1^ d.w.)	(g kg^−1^ d.w.)	(g kg^−1^ d.w.)	(g kg^−1^ d.w.)	(g kg^−1^ d.w.)	(g kg^−1^ d.w.)	(g kg^−1^ d.w.)
H	I	523.29 ± 229.17 c	4.33 ± 0.42 a	72.06 ± 3.22 ab	0.77 ± 0.05 b	7.64 ± 0.41 b	2.26 ± 0.1 b	0.39 ± 0.01 c	0.52 ± 0.11	6.07 ± 1.07
	II	3311.48 ± 148.39 a	3.47 ± 0.37 ab	85.79 ± 4.7 a	0.96 ± 0.1 b	15.59 ± 1.27 a	3.21 ± 0.16 b	0.94 ± 0.02 a	0.8 ± 0.14	14.36 ± 1.75
	Mean	1917.38	3.9	78.92	0.87	11.62	2.73	0.66	0.66	10.21
AQ	I	1565.55 ± 156.84 b	3.94 ± 0.22 ab	57.57 ± 3.65 bc	1.32 ± 0.04 a	16.5 ± 0.71 a	6.09 ± 0.32 a	0.34 ± 0.01 c	0.4 ± 0.09	4.2 ± 0.38
	II	3478.24 ± 321.61 a	2.54 ± 0.29 b	54.24 ± 1.66 c	0.73 ± 0.05 b	15.13 ± 0.15 a	5.3 ± 0.22 a	0.57 ± 0.04 b	0.56 ± 0.08	9.57 ± 0.19
	Mean	2521.89	3.24	55.9	1.02	15.81	5.69	0.46	0.48	6.88
Significance										
S		0.028 **	0.085 ^ns^	0.00 **	0.042 *	0.001 ***	0.00 **	0.00 **	0.132 ^ns^	0.013 ^ns^
C		0.00 **	0.01 **	0.174 ^ns^	0.014 *	0.003 **	0.731 ^ns^	0.00 **	0.079 ^ns^	0.00 **
C × S		0.088 ^ns^	0.443 ^ns^	0.04 *	0.00 **	0.00 **	0.004 **	0.00 **	0.592 ^ns^	0.2 ^ns^

Data represent the mean ± s.e. (n = 3). Different letters indicate significance based on Tukey’s HSD post hoc test. ns, *, **, and *** indicated non-significant and significant effects at *p* < 0.05, 0.01, and 0.001, respectively.

**Table 4:** Photosynthetic pigments content in terms of chlorophyll_(a+b)_, carotenoids, Chl_a/b_ ratio, and SPAD values Genovese basil cv Sanremo plants under two consecutive cuts (35 (I) and at 79 (II) days after planting (DAP)) grown under hydroponics (H) or aquaponics (AQ) conditions.

Treatment		Chl a/b	Chl (a + b)	Total Carotenoids	SPAD Index
System (S)	Cut (C)		(mg g FW^−1^)	(mg g FW^−1^)	
H	I	1.35 ± 0.01 b	1.76 ± 0.04 a	0.22 ± 0.00 c	23.13 ± 0.75 a
	II	1.82 ± 0.04 a	0.95 ± 0.03 c	0.25 ± 0.01 b	13.11 ± 0.88 c
	Mean	1.59	1.35	0.24	18.12
AQ	I	1.78 ± 0.06 a	1.25 ± 0.05 b	0.29 ± 0.00 a	19.41 ± 0.94 b
	II	1.74 ± 0.04 a	1.12 ± 0.1 bc	0.27 ± 0.01 ab	15.97 ± 0.64 bc
	Mean	1.76	1.19	0.28	17.69
Significance					
S		0.005 **	0.024 *	0.00 **	0.611 ^ns^
C		0.001 ***	0.00 **	0.15	0.00 **
C × S		0.001 ***	0.001 ***	0.002 **	0.004 **

Data represent the mean ± s.e. (n = 3). Different letters indicate significance based on Tukey’s HSD post hoc test. ns, *, **, and *** indicated non-significant and significant effects at *p* < 0.05, 0.01, and 0.001, respectively.

## Data Availability

The data are contained within the article.

## Supplementary Materials

The following supporting information can be downloaded at: https://www.mdpi.com/article/10.3390/plants12061355/s1: Table S1: Nutrient concentration in the hydroponic (H) and aquaponic (AQ) systems.
