# Supplementary material for: Hydroponic and Aquaponic Floating Raft Systems Elicit Differential Growth and Quality Responses to Consecutive Cuts of Basil Crop
Source: Plants (Basel). 2023 Mar 17;12(6):1355. doi: 10.3390/plants12061355 (PMC10053589; doi:10.3390/plants12061355)
Supplement: Supplementary file 1 [file plants-12-01355-s001.zip › plants-2194586-supplementary.pdf]

Table S1. Nutrient concentration in the hydroponic (H) and aquaponic (AQ) systems

	**Concentration (mg L^{-1})**							
**System**	**NO_3**	**PO_4**	**K**	**SO_4**	**Ca**	**Mg**	**Na**	**Cl**
H	326.05	25.69	94.79	76.40	57.07	11.32	9.24	5.95
AQ	340.14	11.23	40.23	44.14	74.60	15.01	31.42	52.17
